# Behavior of a Metabolic Cycling Population at the Single Cell Level as Visualized by Fluorescent Gene Expression Reporters

**DOI:** 10.1371/journal.pone.0012595

**Published:** 2010-09-07

**Authors:** Sunil Laxman, Benjamin M. Sutter, Benjamin P. Tu

**Affiliations:** Department of Biochemistry, The University of Texas Southwestern Medical Center at Dallas, Dallas, Texas, United States of America; Texas A&M University, United States of America

## Abstract

**Background:**

During continuous growth in specific chemostat cultures, budding yeast undergo robust oscillations in oxygen consumption that are accompanied by highly periodic changes in transcript abundance of a majority of genes, in a phenomenon called the Yeast Metabolic Cycle (YMC). This study uses fluorescent reporters of genes specific to different YMC phases in order to visualize this phenomenon and understand the temporal regulation of gene expression at the level of individual cells within the cycling population.

**Methodology:**

Fluorescent gene expression reporters for different phases of the YMC were constructed and stably integrated into the yeast genome. Subsequently, these reporter-expressing yeast were used to visualize YMC dynamics at the individual cell level in cultures grown in a chemostat or in a microfluidics platform under varying glucose concentrations, using fluorescence microscopy and quantitative Western blots.

**Conclusions:**

The behavior of single cells within a metabolic cycling population was visualized using phase-specific fluorescent reporters. The reporters largely recapitulated genome-specified mRNA expression profiles. A significant fraction of the cell population appeared to exhibit basal expression of the reporters, supporting the hypothesis that there are at least two distinct subpopulations of cells within the cycling population. Although approximately half of the cycling population initiated cell division in each permissive window of the YMC, metabolic synchrony of the population was maintained. Using a microfluidics platform we observed that low glucose concentrations appear to be necessary for metabolic cycling. Lastly, we propose that there is a temporal window in the oxidative growth phase of the YMC where the cycling population segregates into at least two subpopulations, one which will enter the cell cycle and one which does not.

## Introduction

The budding yeast *Saccharomyces cerevisiae* has long been known to be capable of exhibiting various modes of oscillatory behavior [1]–[6]. When yeast cells are grown to a high density, starved for a short period, and then continuously fed low concentrations of glucose using a chemostat, the cell population becomes highly synchronized and undergoes robust oscillations in oxygen consumption termed yeast metabolic cycles (YMC) [5]–[7]. Such cycles can range anywhere from 40 minutes to over 10 hours depending on the continuous glucose concentration. They consist of phases of rapid oxygen consumption (oxidative) that alternate with phases of minimal oxygen consumption (reductive). A variety of growth and metabolic parameters such as budding index, storage carbohydrate content, ethanol levels, and carbon dioxide production have been observed to oscillate as a function of such cycles, although not necessarily in phase with the dissolved oxygen oscillation [5], [8]–[10].

From comprehensive gene expression studies, we previously determined that over half of yeast genes (>57%) are expressed periodically during long-period, 4–5 hour cycles [5]. Gene products with functions associated with energy and metabolism and those localized to the mitochondria tend to be expressed periodically. Moreover, genes that encode proteins with a common function tend to exhibit similar temporal expression profiles. Analysis of this YMC expression dataset revealed three superclusters of gene expression, which we used to define three major phases of the YMC: OX (oxidative, respiratory), RB (reductive, building), and RC (reductive, charging) [5]. Different categories of genes peak during each phase, and cells traverse each of these three phases in every metabolic cycle. The OX phase represents the peak of mitochondrial respiration and is associated with a rapid induction of ribosomal genes and other genes involved in growth. Cell division and the upregulation of genes that encode mitochondrial proteins occur during the RB phase, when the rate of oxygen consumption begins to decrease. In the RC phase, many genes associated with stress and starvation-associated responses (e.g., ubiquitin-proteasome, vacuole, autophagy, heat shock proteins, detoxification enzymes) are activated prior to the next OX phase [5].

Studies of these cycles have revealed the changes in metabolism that occur during the life of a yeast cell and provided significant insight into how a number of important cellular processes might be coordinated with metabolism [5], [11]. However, it is not known whether such metabolic cycles might occur in single individual cells in the wild, in the absence of a glucose-limited, steady-state growth environment maintained by the chemostat. Moreover, in each permissive window of the YMC, approximately half of the cell population initiates the cell division process [5], raising questions about the metabolic behavior of the remaining cells that are not dividing. Using quantitative *in situ* hybridization methods, a recent study has provided evidence that such metabolic cycles occur in single cells in unsynchronized chemostat cultures under either glucose or phosphate limitation, by examining the correlation of pairs of probes targeting genes from either the same or distinct phases [12]. In a separate study, a luciferase reporter has been successfully developed to monitor gene expression of the YMC population as a whole [13]. However, gene expression reporters that enable tracking the YMC in single cells in real-time have not yet been developed, nor have large populations of metabolically cycling cells been visualized with phase specific reporters.

In this study, we utilized a series of fluorescent gene expression reporters designed to peak during distinct phases of the YMC to investigate the behavior of individual cells within metabolically cycling cell populations. In addition, we transferred cycling cells from a chemostat to a microfluidic device that enables real-time imaging of live single cells under varying nutrient conditions to determine the resulting consequences on such metabolic cycles. These experiments have revealed interesting and unexpected behaviors of single cells within cycling populations and suggest the presence of heterogeneous subpopulations of cells that may intimately cooperate to achieve the striking synchrony of the entire population as visualized by macroscopic dissolved oxygen measurements.

## Results and Discussion

### Cells in the YMC are sub-grouped into dividing and non-dividing cells

**Figure 1:**
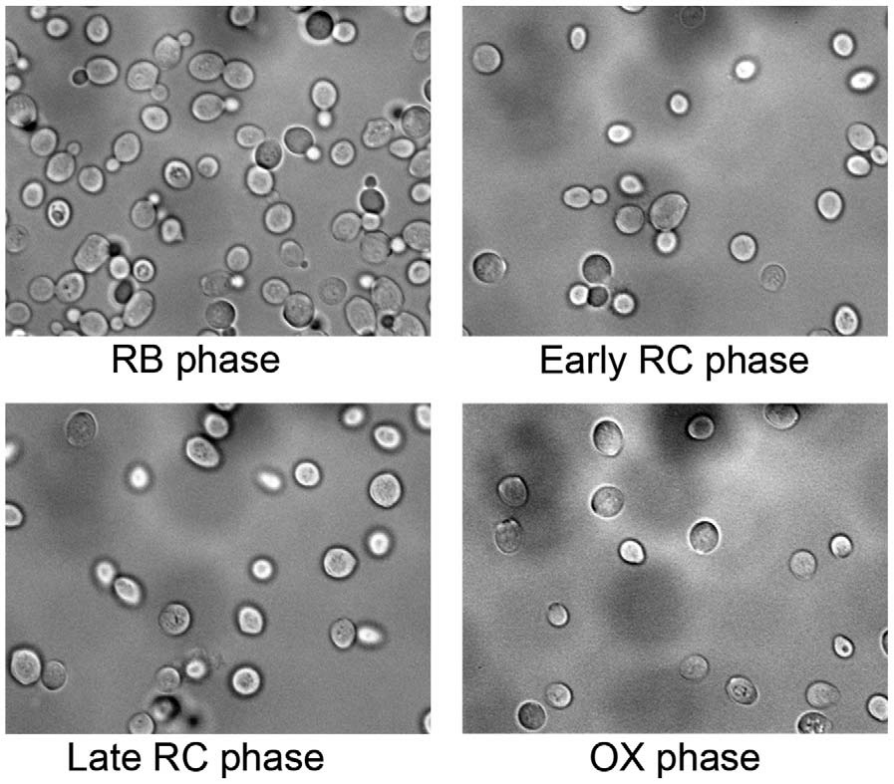
Dividing and non-dividing cells in the RB phase of the YMC. Brightfield images of diploid yeast cells undergoing the YMC from the Reductive-Building phase, the early Reductive-Charging phase, the late Reductive-Charging phase, and the Oxidative phase. Cell division for about half of the total cell population occurs exclusively during the RB phase, while no cells divide during the RC and OX phases.

Using microscopic visualization, we aimed to better understand the YMC by examining the behavior of single cells within the cycling population. Figure 1 shows diploid yeast cells from different phases of the YMC. In samples of cycling cells, dividing (budding) cells first appeared at the end of the OX/beginning of RB phase, and nearly all cell division initiated exclusively in the RB phase (Figure 1A), in agreement with previous observations [5]. The cells also appeared to be highly synchronized, and all cells with growing buds were at a similar stage of cell division. Importantly, in the RC phase there were no budding cells observed even in large populations (Figure 1B, 1C). Interestingly, only about half of all the diploid cells within this population entered cell division in each RB phase of the YMC, while the other half of the cell population did not divide (267 budding cells in a total sample pool of 581 cells collected from late RB phase, ∼46% budding cells). These observations suggest that while the basic cell division processes are tightly temporally regulated as a function of the YMC, there appears to be a significant heterogeneity within the population of cells itself. The cells within the YMC appear to fall into two broad sub-populations of cells; those that actively divide in each RB phase of the YMC, and those cells that appear to not be actively participating in cell division.

### GFP reporters correlate with gene mRNA expression but cells undergoing the YMC exhibit heterogeneity in reporter expression

**Figure 2:**
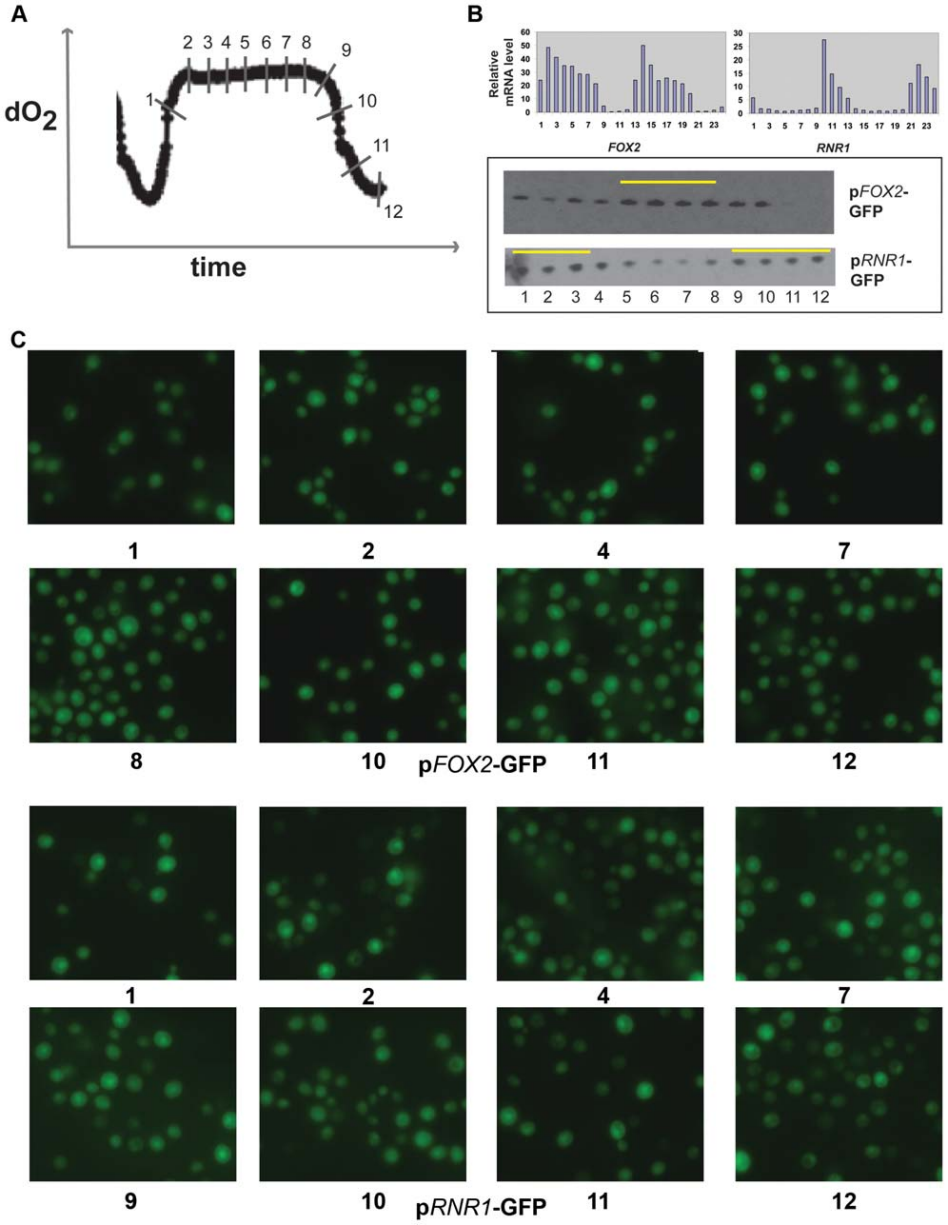
RB or RC phase GFP reporter expression across the YMC tracks with gene mRNA expression. (A) Samples from haploid cells expressing single GFP reporters of either an RB phase or an RC phase gene (p*RNR1*-GFP or p*FOX2*-GFP) were collected from across the YMC at the time points indicated. (B) mRNA levels of selected RB phase and RC phase genes (*RNR1* and *FOX2*) across two consecutive cycles as determined previously using microarray analysis [5], and western blots showing GFP protein expression levels (of the reporters p*RNR1*-GFP and p*FOX2*-GFP) in separate cell samples collected from corresponding time points (indicated in panel A) across the YMC. The time points showing the highest reporter expression are highlighted by a yellow line. (C) Panels show images of cells from the YMC expressing p*FOX2*-GFP at the indicated time points. (D) Panels show images of cycling cells expressing p*RNR1*-GFP at the indicated time points. Loading controls for panel B are shown in Figure S1.

Our previous work showed that mRNA expression profiles of different genes are very tightly regulated within different phases of the YMC, correlating to the temporal separation of key biological processes [5]. However, from earlier studies it was not clear (i) how tightly these mRNA expression profiles correlate with mRNA translation and protein production and (ii) how homogeneous or heterogeneous the cell populations within the YMC were. In order to address these questions, we examined GFP reporters for genes from the RB or RC phases across the YMC. In Figure 2, the reporter expression from two different haploid yeast strains tagged either with a GFP reporter for *RNR1* (p*RNR1*-GFP, RB phase reporter) or *FOX2* (p*FOX2*-GFP, RC phase reporter) are shown. Similar experiments were also performed for OX and RC phase GFP reporters made for genes such as *ATO3*, *GND2* and *DBP2* (not shown), and the trends observed were very similar to the reporters shown here. Importantly, at the GFP protein level (as measured by quantitative immunoblotting), the OX, RB and RC phase reporter protein expression peaked at times close to when the corresponding gene mRNA expression was highest (mRNA data from [5]) (Figure 2B and Figure S1). These results suggest that the actual translation of the mRNA and protein synthesis follow the mRNA expression profiles across the YMC for these genes. A few reporters were made for mitochondrial RB phase genes, but these reporters were unsuccessful and did not show detectable expression (not shown). Surprisingly, the GFP signal for all reporters tested remained fairly strong across the YMC, and the quantitative differences seen by immunoblotting were not as apparent when visualizing individual cells within populations (Figure 2C and not shown). It is possible that the persistence of the fluorescence is due to the known extended stability of GFP [14], [15]. The half-life of the GFP protein in cycloheximide-treated cells undergoing metabolic cycles was ∼4.5 hours (Figure S2), which is approximately the duration of one complete cycle in our culturing conditions. While this is longer than the mRNA cycles of typical phase-specific genes, it was sufficient to observe oscillations of phase-specific reporters between independent cycles as assayed on the population by Western blot (Figure 2B). Moreover, this measured half-life is shorter than the extended half-lives of GFP measured in typical systems [16], [17]. Similar reporters made with a destabilized GFP variant [16], [17] did not behave consistently well and appeared to exhibit unusually short half-lives (not shown).

Importantly, in all the GFP reporter strains tested, only about half the total cell population appeared to maintain significant expression of the reporter across the YMC. While changes in GFP protein level within this population were not visually obvious across different time points in the YMC, it was apparent that about half the cells exhibited only basal expression of GFP. This was true for the RC as well as OX reporters tested (Figure 2C and not shown). This is perhaps consistent with our earlier observation that half the cell population does not divide in a given metabolic cycle. However, it also suggests an unanticipated degree of complexity and heterogeneity within the population of cells despite the observation that the population as a whole is cycling normally based on macroscopic dissolved oxygen measurements.

### Dual-fluorescent reporters of different YMC phases

**Figure 3:**
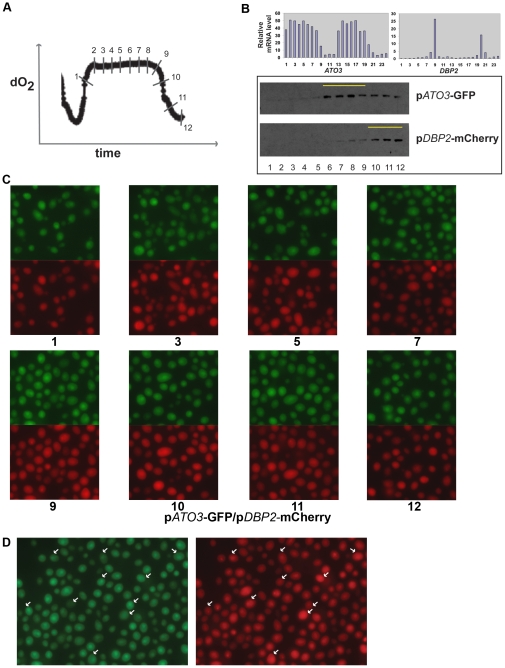
Dual reporters representing different YMC phases show separation of peak expression. (A) Samples from diploid cells expressing two reporters, an mCherry reporter of an OX phase gene (p*DBP2*-mCherry) and a GFP reporter of an RC phase gene (p*ATO3*-GFP) were collected from across the YMC at the time points indicated from 1-12. (B) mRNA levels of *DBP2* and *ATO3* across two consecutive cycles of the YMC as determined previously [5], and western blots showing reporter GFP or mCherry protein levels across the YMC. The time points with highest reporter expression are highlighted by a yellow line. (C) Panels show images of cycling cells expressing p*DBP2*-mCherry and p*ATO3*-GFP at the indicated time points. (D) A larger field of cells from time point 9, showing the expression levels of the two reporters within the same cell. A partial selection of cells showing opposite expression of red or green reporters within the same cell are highlighted with white arrows. Loading controls for panel B are shown in Figure S1.

In order to further understand the subpopulations of cells within the YMC, we created diploid yeast strains that simultaneously expressed both an OX phase reporter using mCherry (p*DBP2*-mCherry) and an RC phase reporter using GFP (p*ATO3*-GFP). The OX phase reporter should be representative of the growth phase when cellular metabolic activity and respiration is highest. These dual-reporter strains allowed us to directly compare expression profiles of different phase reporters within the same cell and assess whether cells were in either a “rapid growth” or “slow growth/stationary” state, over the YMC. Quantitative immunoblots (Figure 3B) show that the peak protein expression of RC phase reporters and the OX phase reporters are temporally separated between the phases, and the peak protein expression for each reporter gene occurs after the peak in mRNA levels of the actual genes. Similar results were seen with other reporter sets (not shown). However, these changes in protein level were once again not as visually obvious when imaging individual cells within populations across the YMC (Figure 3C). Instead, these dual-reporter strains suggest that there is significant heterogeneity even within cells that are expressing the reporters. As observed earlier, approximately half the cells only show low/basal expression of OX and RC reporters. With the remaining cells, we observed fairly strong expression of OX or RC phase reporters (Figure 3C). Importantly, in many cells we observed clearly contrasting and opposite expression of OX and RC reporters (Figure 3D, which uses time point 9 as an illustrative example), where the OX reporter expression is relatively low in cells with high RC reporter expression and vice versa. In the remaining cells that show expression of the reporters, the levels of RC and RB reporters are not as obviously opposite, but appear to be similar. Thus, we suggest that although there is very tight regulation of mRNA expression of specific genes as determined by microarray analysis, and very tight control of cell division within the YMC, this temporal sequence of events is not easily captured via conventional use of fluorescent reporters, perhaps partiallyly due to the extended half-life of GFP/RFP [16], [17] (Figure S2). Though the YMC is extremely robust, there appears to be a sophisticated organization and communication between subgroups of cells within the cycling population.

### Cells committed to division show temporal accumulation of both OX and RB phase reporters

**Figure 4:**
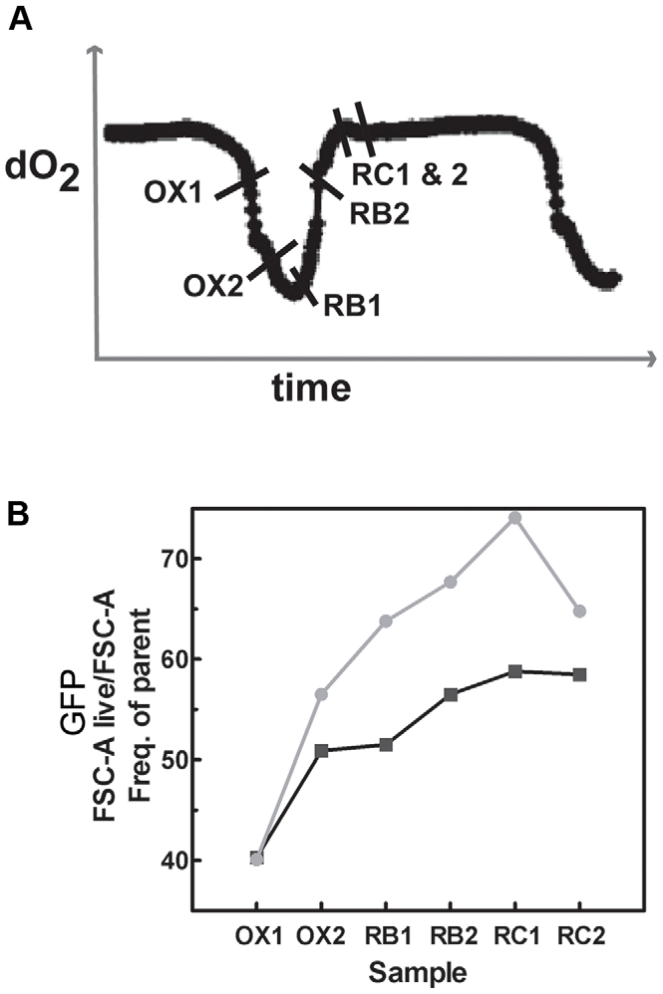
Cells committed to entering the OX phase show concurrent increased RB phase reporter expression. Flow cytometry analysis of cells from the YMC expressing an OX phase growth reporter and a RB phase cell cycle reporter showed an increase in the number of cells expressing the cell cycle reporter within cells that have high OX phase reporter expression. (A) Schematic illustration showing the time points when cells expressing a cell cycle/RB phase reporter (p*RNR1-*GFP) and an OX phase reporter (p*DBP2-*mCherry) were collected. Similar time points were collected from two independent cycles. (B) A plot with two independent data sets showing the expression of the GFP reporter (p*RNR1-*GFP) within cell populations sorted for highest mCherry expression of a OX phase reporter (p*DBP2-*mCherry), at different specified times of the YMC. The two lines (grey/grey circles and black/black squares) represent data from two independent cycles.

The p*DBP2-*RFP reporter was designed to represent an OX phase growth reporter while the p*RNR1-*GFP reporter was designed to represent an RB phase cell cycle reporter. However, the p*RNR1*-GFP reporter unexpectedly peaked in expression at about the same time as the p*DBP2-*mCherry reporter (Figures 2B, 3B). We constructed a single yeast strain that expresses both of these reporters, in order to determine if there was any correlation in expression between these two reporters within a single cell. Using flow cytometry, we sorted cells into quadrants based on highest GFP and mCherry expression, and examined the expression of the p*RNR1-*GFP reporter in cell populations that had entered the OX phase and showed the highest p*DBP2-*mCherry expression. Samples were collected at the time points shown in Figure 4A, fixed and subsequently analyzed using flow cytometry. The results of this experiment were plotted on a frequency of parent vs. sample time plot, which shows the frequency of cells expressing the GFP reporter within the population of cells with highest mCherry expression (Figure 4B), with data from two independent metabolic cycles. The data consistently and clearly showed a steady and significant increase in the population of cells expressing high GFP levels within the cell population already committed to the OX phase (high mCherry expression). These data strongly support the hypothesis that the cells which express OX growth phase genes will be the ones that subsequently enter cell division and express cell cycle genes such as *RNR1*. The data further support the idea that once committed to growth, the OX and RB gene expression programs ensue.

### Low glucose levels are necessary to observe the YMC

**Figure 5:**
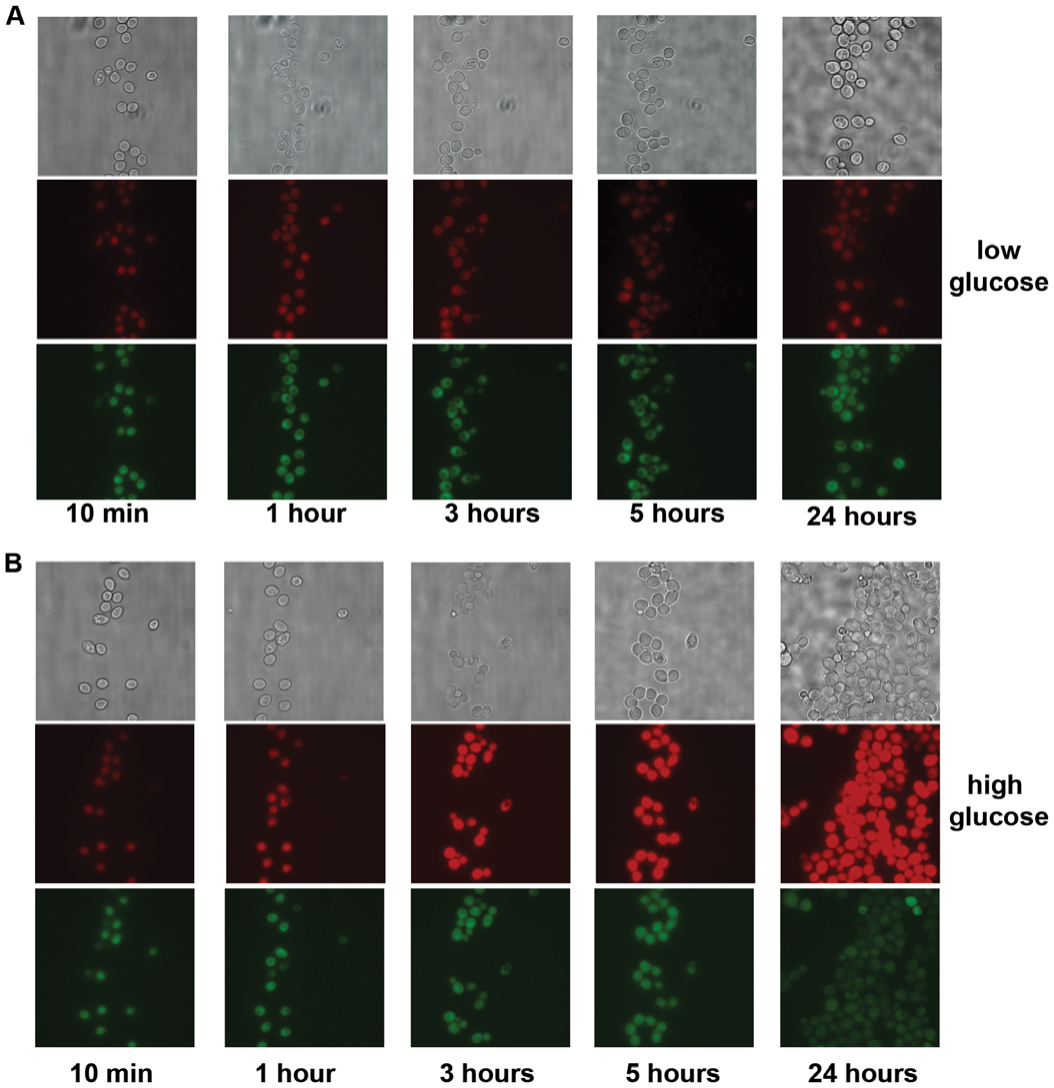
Low glucose levels are necessary for maintaining metabolic cycles. Images of live yeast cells expressing OX and RC phase reporters continuously growing in a microfluidics chamber show the importance of low glucose levels for maintaining metabolic cycles. (A) Cycling cells expressing an RC reporter (p*ATO3-*GFP) and an OX reporter (p*DBP2-*mCherry) were transferred to a microfluidics chamber, grown under low glucose concentrations and imaged at the specified times. (B) Cycling cells expressing the two reporters were transferred to a microfluidics chamber, grown under high glucose concentrations and imaged at the specified times.

The use of dual-reporters enabled us to obtain qualitative information about the general behavior of cells undergoing the YMC. However, the cells undergoing the YMC were grown in controlled chemostat cultures. The chemostats enabled the simulation of an environment where cells could grow to high densities, yet were kept alive and continued to grow under continuous, nutrient-restricted (and not nutrient-depleted) conditions. We initially wondered how important glucose limitation alone was for metabolic cycling, or if the relative cell density (as well as secreted or synthesized factors) played a role in cell synchronization. In attempt to continuously visualize live yeast cells undergoing metabolic cycling, we used a dynamic cell culture platform (CellASIC) that contains a microfluidic chamber where cells could grow and be fed with varying nutrients from a feed well. The microfluidic plates contain two separate flow units, which can switch between two different solutions that allow continuous or interrupted feeding. Dual-reporter-expressing cells undergoing metabolic cycles were taken from the chemostat and rapidly transferred to the microfluidic chamber. Here, using the two flow units, the cells were fed continuously either with depleted media containing low glucose concentrations (0.001% w/v glucose), or with a typical high glucose medium (containing 1% w/v glucose). The cells growing within these chambers were imaged over 24 hours. Figure 5 shows the results of reporter gene expression in these cells when transferred from the chemostat into the microfluidic chamber under these different conditions. Under low glucose conditions, the cells continued to divide approximately every 3–4 hours, similar to division times observed in the chemostat. Not surprisingly, both reporters (OX and RC phase) showed significant expression at the beginning of growth within the chamber. Interestingly, the cells growing with continuously available but low glucose continued to show steady expression of the OX and the RC phase reporters for over 20 hours, and maintained fairly synchronized cell division (Figure 5). Under these conditions, the cells appeared to remain somewhat synchronous over this duration of time, dividing at reasonable intervals (Figure 5A). In stark contrast, when metabolically cycling cells were grown in the microfluidic chamber and introduced to high glucose concentrations, the OX reporter expression increased rapidly and dramatically over time, while the RC reporter expression dropped steadily, to reach basal levels after 6–8 hours. The cells lost all synchronized behavior, and began to divide rapidly and randomly during this time (Figure 5B).

These results show the apparent requirement for low glucose levels to enable metabolic cycling. While low glucose itself appears to be necessary for this phenomenon, it may not alone be sufficient, and other macro-nutrients (in particular phosphate or nitrogen) may play a role in enabling cycling [12]. Importantly, cells growing in high glucose quickly lost any metabolic synchronization and grew unrestrictedly, abandoning the stringent “quality control” and “survival” processes which the genes that peak in the RC phase typically enable. Even under low glucose conditions, the cells growing in the microfluidic chamber eventually lost synchronization of cell division, and the expression of the OX phase reporter gradually increased, while that of the RC phase reporter slowly decreased beyond 30 hours of growth, depending upon the reporter used (not shown). These observations are consistent with the idea that RC phase genes are typically expressed under slower growth rates, while OX phase genes are upregulated in response to higher growth rates [18], [19]. While the microfluidic chamber allows continuous culture of cells with constant feeding of media at fixed rates, there are two key differences between the microfluidic chamber and the chemostat culture. The first is the difference in the cell density and cell population distribution. The microfluidic chamber utilized traps cells based on their size. Hence, the three different grids in the chamber separate three different populations of cells based on size. Additionally, the cell density within the chamber may be different from the density within the chemostat, with individual cells separated from each other, and with populations separated by size. Secondly, the volume within the chamber is small, and the media within it is constantly replaced. Thus, while the glucose concentrations remain low within the microfluidic chamber, it is possible that the cycling cells are not at the same density as they are in chemostat cultures, and are no longer in physical communication with other cells, including the cells that do not divide. Therefore, it is reasonable to speculate that additional cell communication factors that are secreted either by dividing cells themselves or the non-dividing cells in each cycle may be required for the community to maintain synchronous metabolic cycles for extended periods of time. However, low glucose levels (along with restricted levels of other macro-nutrients) appear to be necessary for metabolic cycling. Factors such as H_2_S, acetaldehyde and ethanol have been implicated in the establishment of synchrony in yeast cells [20], [21], and they or other as yet unidentified factors may be important for setting or maintaining the YMC. These studies also suggest the suite of OX phase genes are expressed at higher levels during log phase growth, while the expression of RC phase genes is repressed or minimal. Consistent with this idea, many RC phase gene products are present at very few molecules per cell in log phase [22]. The suite of RC phase genes may be induced upon entry into stationary phase as growth rate slows [19]. We predict the RC gene expression program functions to enhance cell survivability and that sufficiently low glucose conditions are capable of inducing this program.

### Reporter expression suggests differences between cells in the RC phase and stationary phase quiescent cells

**Figure 6:**
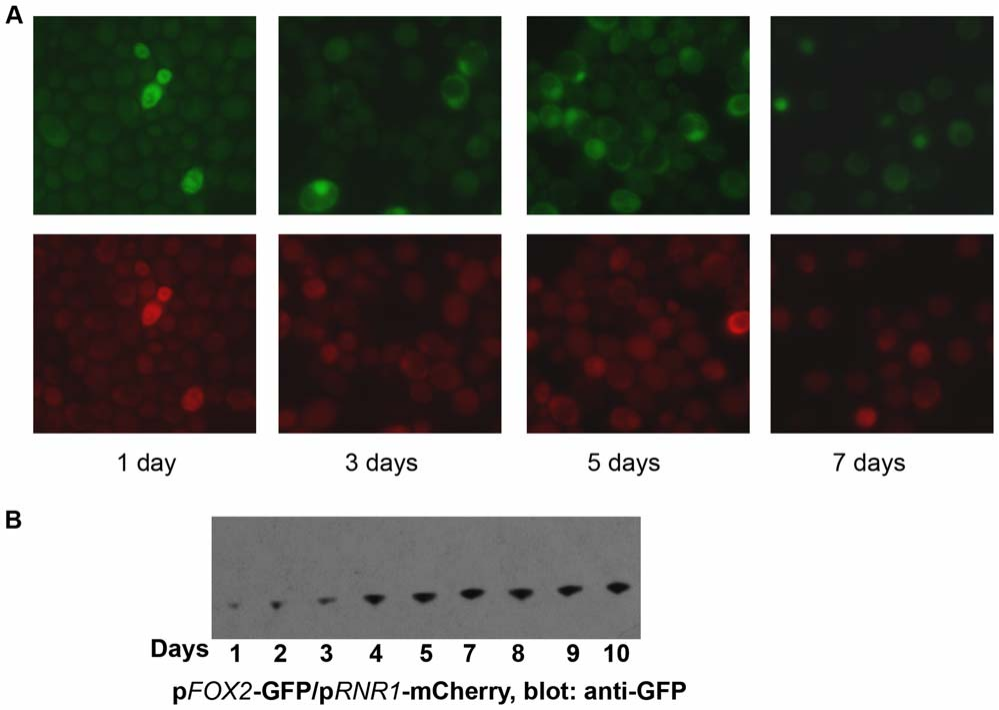
Stationary phase quiescent cells show low levels of RB and RC phase reporter expression. (A) Expression of RB phase (p*RNR1-*mCherry) or RC phase (p*FOX2-*GFP) reporters in yeast cells growing in batch cultures in synthetic defined glucose medium and subsequently transferred to water over a period of ten days. (B) Western blot showing the expression of the RC phase reporter (p*FOX2-*GFP) from batch cultures, as a function of time spent in stationary phase. The RB phase reporter (mCherry/RFP) could not be detected in these cells by Western blot (to detect mCherry) for the same samples as shown. Similar data was obtained for all other RC/OX or RC/RB phase dual color reporters tested. Loading controls for panel B are shown in Figure S3.

In previous studies, we showed that cells in RC phase exhibit hallmarks of quiescent cells, based on the criteria of individual cell density [23]. Using the dual-reporter strains, we observed the expression of RB and RC phase reporters in cells grown under typical “quiescent” culture conditions for a period up to 10 days. Various dual-reporter strains (Figure 6, and not shown) were grown in synthetic defined glucose medium to saturation, and were allowed to remain in glucose-depleted SD medium over a ten day period (Figure 6) or subsequently transferred to water and shaken (not shown). In both cases, and for three different reporter sets, the results were largely similar. Interestingly, these results appeared different from those we had observed in cells undergoing metabolic cycles. In the cells grown to stationary phase under typical batch culture conditions, the overall expression of RC phase reporters increased slightly over time but remained very low (Figure 6). This is in contrast to our description of the cells in the RC phase where we observed more significant expression of the RC phase reporters, representing numerous genes that are known to enable stringent protein quality control processes and enhance cell survival under various stresses. Furthermore, the RB phase cell cycle reporter, which is strongly expressed in cells in the log phase in high glucose concentrations (Figure 6B and not shown) showed only a low/basal expression in these stationary phase quiescent cells in this duration of time. Thus, cells in the RC phase appear to be in a state where they undergo extensive cellular quality control processes that actively prepare the cells to become “primed” for another round of cell division. It is possible that the RC phase can be likened to “early stationary phase”, where cells are adapting to nutrient and starvation stresses but remain primed towards entering growth under favorable conditions, and that upon prolonged incubation of cells in depleted media gene expression is drastically reduced as they enter a more dormant state. Indeed, cells in extended starvation/stationary phase exhibit very low rates of transcription and translation [24].

**Figure 7:**
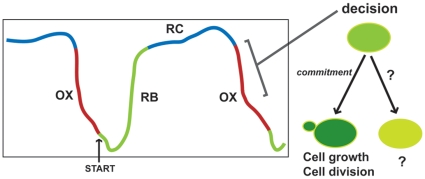
A proposed model describing two subpopulations within the YMC. The schematic illustration shown for the YMC suggests a decision point where cells irreversibly commit to entering cell growth and division, separating the population into dividing cells and non-dividing cells.

We speculate that the RC phase is a state where cells induce a gene expression program to maximize survivability under nutrient-poor conditions and further prepares cells for regrowth once conditions improve. This would enable the cells to continue to persist in the most optimum manner possible under stringent conditions. We also speculate that it is in the RC or early OX phase that key decisions for the community, such as which cells will divide and which will not, are made. In more ways than one the RC phase appears to be biologically interesting and complex, and future studies will reveal the logic of the cellular processes and activities that dominate the RC phase. Figure 7 proposes a model showing this decision point in the late RC phase or early OX phase where particular cells within the YMC commit irreversibly to growth and division and subsequently express OX and cell cycle genes. A particularly interesting future aim will be to understand the role of the cells that do not commit to growth and division in each cycle.

There remain some significant limitations to using fluorescent reporters to probe the YMC. Although the mRNA of the reporters are under the control of the same promoter and 3′UTR regulation as the endogenous gene, thereby making it likely that reporter expression matches the actual phase-specific genes, it is possible that the reporters do not fully reflect the inherent stability of the respective proteins. GFP and mCherry proteins are known to have inherent stability (Figure S2) [14], [16], [17], [25]. This extended stability of GFP/RFP is a limitation in obtaining a fully quantitative understanding of phase-specific gene dynamics. Fluorescent reporters may also exhibit substantial maturation times [25]. However, using a p*RNR1-*GFP reporter we observed an increase in GFP expression concurrent with bud emergence, suggesting there is not a significant delay due to fluorophore maturation in the YMC. Reporters containing destabilizing sequences have been constructed [16], [17], however in our hands the properties of the few reporters tested made them unsuitable for use in the YMC because they are too rapidly degraded (not shown). The use of specially-designed, destabilized fluorescent reporters may be required for future studies. In addition, it remains possible that there are determinants of mRNA expression, stability and translation within the gene coding sequences that may not be fully recapitulated using the reporter design in this study. There are also likely to be other factors influencing mRNA expression and stability such as chromatin-based determinants that may be lost when integrating reporters at an exogenous locus. Hence, some of these issues must be addressed carefully in future studies and particularly when using reporters in other experimental contexts.

Nevertheless, using these reporters we have been able to obtain some insights into the YMC. We conclude by summarizing our observations and raising further questions about the YMC. Within the cycling cell population, there exists significant heterogeneity in the expression of reporters representing the various YMC phases, suggesting the presence of distinct subpopulations of cells. If so, these observations suggest an inherent robustness within the YMC, which allows a degree of variability between such subpopulations while still maintaining highly synchronized cell division and optimal utilization of limited nutrients. Importantly, there appears to be a commitment or decision point for these cells that occurs in late RC or early OX phase, and if the commitment has been made, a cell will undergo growth, division and complete the sequential, temporally regulated expression of OX and RB phase genes (Figure 7). What factors allow this simultaneous synchronization and heterogeneity? Secondly, what are the cells that are not dividing doing during each cycle? Do they remain in a metabolically dormant state, do they secrete factors that contribute to cycling, or do cells take turns to divide, in alternate cycles? Future studies focused on the characterization of the specific subpopulations of cells will provide insight into their roles. Whatever they may be doing, the gene expression and metabolic programs of these cells do not appear to obscure gene expression and metabolite measurements of the population as a whole [5], [11]. Some of these questions require new techniques to be developed in order to address them, including methods to culture cells in microfluidic chambers that maintain cell density and communication, reporters that more accurately reproduce the actual behavior of the genes they represent, as well as tracking individual cells within the chemostat over time. The work presented here is a step towards further understanding this fascinating biological phenomenon that may provide insights into many biological processes including cell growth, cell division, metabolism, and the logic of cell-cell communication within complex, intricate microbial communities.

## Materials and Methods

### Yeast strains

The prototrophic CEN.PK strain background was used in all experiments [26].

### Reporter construction

The reporters were constructed using GFP or mCherry sequences under the regulation of specific gene promoters, as well as their 3′ untranslated regions (3′UTR). The entire GFP (EGFP) or RFP (mCherry) sequences were amplified by PCR and cloned into the multiple cloning site of the p417cyc vector. ∼500 nucleotides upstream of the start codon of the various reporter genes used were directly PCR-amplified from *S. cerevisiae* genomic DNA. Similarly, 300 nucleotides downstream of the stop codon of the reporter genes were directly PCR-amplified from genomic DNA. These sequences were then inserted upstream and downstream of the GFP or RFP sequences in the respective p417cyc backbone vector. This complete 5′UTR-reporter-3′UTR sequence was then PCR amplified and transferred to the M4297 vector (HO-poly-KanMX-HO) [27], which can be used to chromosomally integrate genes in the non-essential HO locus in budding yeast. The reporters were subsequently transformed into haploid CEN.PK and integration of the complete cassette at the HO locus was confirmed by PCR amplification. For dual-reporter strains, appropriate reporters expressed in opposite mating type strains were mated, diploids selected, and the expression of both reporters in the diploid cells was confirmed by fluorescent microscopy. A list of reporter strains made and used in this study is shown in Table 1.

**Table 1:** List of reporter strains used in this study.

Reporter name	YMC phase	CEN.PK diploid/haploid
p*DBP2*-GFP	OX	haploid
p*RNR1*-GFP	RB	haploid
p*ATO3*-GFP	RC	haploid
p*FOX2*-GFP	RC	haploid
p*RNR1-*mCherry/*FOX2-*GFP	RB/RC	diploid
p*DBP2*-mCherry/*ATO3*-GFP	OX/RC	diploid
p*DBP2*-mCherry/p*RNR1*-GFP	OX/RB	diploid
p*GND2-*GFP	RC	haploid

### Yeast cell growth and culture

Yeast cells were grown in batch culture using standard defined, complete synthetic yeast medium with 1–2% glucose or YPD medium. Yeast cells were grown in chemostat cultures using semi-defined medium as described earlier [5]. Cells growing in the fermentor were also rapidly transferred to a dynamic microfluidic cell culture chamber (Y2 plates, CellASIC), and grown within these chambers under different media conditions. The Y2 dynamic microfluidic cell culture (CellASIC) chamber allowed cells to be switched between low and high glucose media, and fed continuously for at least 24 hours. The “low glucose media” used media taken from the fermentor, from which the growing cells were removed (by centrifugation and filtration through a 0.2 µ filter), and glucose was added back to a final concentration of 0.001% (w/v). High glucose medium contained the same fermentor media (with cells removed), but with glucose added back to a concentration of 1% (w/v). The respective media were constantly flowed into the chamber at a rate of 3.5–4 psi (7–8 µL/hour). For stationary phase quiescence experiments, cells were grown in complete synthetic defined medium containing 2% glucose (SD), and subsequently transferred to tubes containing water and maintained in these (with daily water replacement) for 10 days, or grown in SD and allowed to remain in nutrient-depleted medium for 10 days.

### Imaging of yeast cells

For imaging of cells from various YMC phases, samples of cells were collected, fixed immediately in 4% paraformaldehyde solution for 20 minutes, spun down, washed 1X in phosphate buffered saline and resuspended in 40 mM phosphate, 1.2 M sorbitol. These cells were placed on poly-L-lysine coated glass slides, allowed to adhere, excess cells were removed, a drop of fluorescent mounting medium (Vectashield, Vector laboratories) was added, and the cells were covered with a glass cover-slip and sealed with nail polish. The slides were stored at −20°C for 2–3 days, and imaged on a Nikon Eclipse 90i microscope. All slides were imaged for the same duration of time, using the same gain and other imaging parameters. Live cells growing within the dynamic microfluidic cell culture chamber were imaged using an Applied Precision deconvolution microscope (DeltaVision) at the UT Southwestern Live Cell Imaging Core Facility.

### Cell sample collection, lysis and immunoblot methods

Typical metabolic cycles were of duration of ∼4–5 hours. Twelve samples for microscopic visualization or immunoblots analysis were collected at fixed time points of 20–28 minutes (depending upon the duration of the cycle) to cover one entire metabolic cycle. For immunoblot analysis, a constant OD of cells from each time point (5 OD units) were collected at the indicated time points, spun down immediately at 15000 g/30 seconds, the cell growth medium was removed and the cell pellet was frozen in liquid nitrogen. Samples were subsequently lysed by bead-beating in 300 µl of Laemmli sample buffer, boiled, and 5 µl of each sample was loaded onto 4–12% Bis-Tris gradient gels (Invitrogen), resolved, and transferred onto nitrocellulose membranes and the reporter protein levels were detected by standard western blotting procedures using an anti-GFP primary antibody (Roche) or an anti-RFP primary antibody (ChromoTek), and HRP-conjugated secondary antibodies against mouse (Bio-Rad) or rat (Jackson Labs) antigens. The mRNA expression profiles data shown for the specified gene mRNAs were obtained from the list of periodic transcripts database described earlier [5], and plotted using Microsoft Excel (Microsoft Corp., WA).

### Flow cytometry and data analysis

Cell samples were collected from metabolically cycling cells expressing the p*RNR1*-GFP reporter and the p*DBP2*-mCherry reporter (RB and OX phase reporters) at the OX, RB and early RC phases, and the cells were fixed as described earlier. The cells were then washed and resuspended in FACS buffer containing phosphate buffered saline containing 25 mM Hepes pH 7.5 and 2 mM EDTA. Aliquots of cells (1×10^7^) were incubated with DAPI for 20 minutes, washed and resuspended in FACS buffer, and cells were analyzed on a BD LSR II flow cytometer. Data analysis was carried using the FlowJo software (TreeStar, Ashland, OR), and the data plotted using the GraphPad Prism version 5 software (GraphPad Software, San Diego, CA).

## Supporting Information

Figure S1 Loading controls for Figures 2B and 3B. (A) Coomassie-stained gels loaded with lysed samples of cells expressing the single GFP reporters, pRNR1-GFP or pFOX2-GFP, shown in Figure 2B. Equal amounts of these samples were loaded in the gels/Western blots shown in Figure 2B. (B) Coomassie-stained gels with equal volumes of lysed samples collected from diploid cells expressing the dual reporters, pATO3-GFP and pDBP2-mCherry shown in Figure 3B. Quantitative Coomassie blue-stained gels were preferred as protein loading controls (over Western blot-based loading controls using housekeeping genes such as actin or GAPDH) since the mRNA of many such genes oscillate significantly across the YMC.
(2.57 MB TIF)

Figure S2 Half-life of GFP in the continuous culture conditions used to observe the YMC. (A) 40 µg/ml cycloheximide was added to metabolically cycling diploid yeast cells expressing pATO3-GFP at the time point indicated. (B) Equal amounts of cells (10 OD600 units) were collected at each time point indicated, lysed in sample buffer (with protease inhibitors) by bead beating, and subsequently resolved and detected as described in the Materials and Methods section. (C) Band intensities corresponding to GFP protein levels were quantified from the blot image using the ImageJ program, and the GFP half-life under these conditions was calculated from this data. The half-life for mCherry (for an mCherry reporter, pDBP2-mCherry) was nearly identical to that of GFP (not shown). The cycloheximide treatment was irreversible and stops metabolic cycles, and the cells in the chemostat did not recover.
(0.83 MB TIF)

Figure S3 Loading control for Figure 6B. Coomassie-stained gels loaded with equal amounts of the samples shown in the gels/western blots displayed in Figure 6B, obtained from quiescent batch cultures of yeast cells expressing GFP/mCherry reporters.
(1.10 MB TIF)
