# Supplementary figures and images for: Behavior of a Metabolic Cycling Population at the Single Cell Level as Visualized by Fluorescent Gene Expression Reporters
Source: PLoS One. 2010 Sep 7;5(9):e12595. doi: 10.1371/journal.pone.0012595 (PMC2935372; doi:10.1371/journal.pone.0012595)

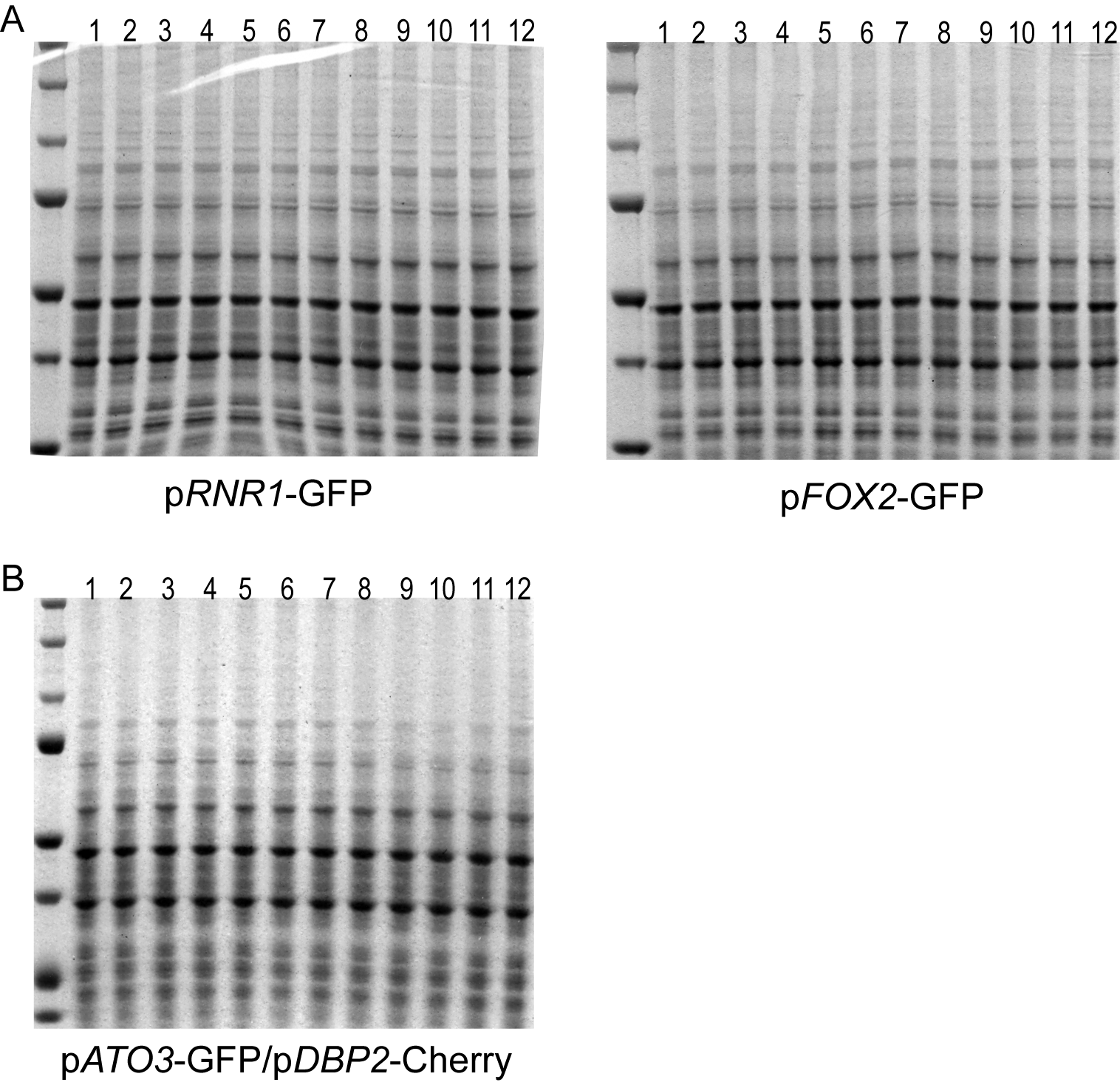

Supplement: Figure S1 — Loading controls for Figures 2B and 3B. (A) Coomassie-stained gels loaded with lysed samples of cells expressing the single GFP reporters, pRNR1-GFP or pFOX2-GFP, shown in Figure 2B. Equal amounts of these samples were loaded in the gels/Western blots shown in Figure 2B. (B) Coomassie-stained gels with equal volumes of lysed samples collected from diploid cells expressing the dual reporters, pATO3-GFP and pDBP2-mCherry shown in Figure 3B. Quantitative Coomassie blue-stained gels were preferred as protein loading controls (over Western blot-based loading controls using housekeeping genes such as actin or GAPDH) since the mRNA of many such genes oscillate significantly across the YMC. (2.57 MB TIF) [file pone.0012595.s001.tif]

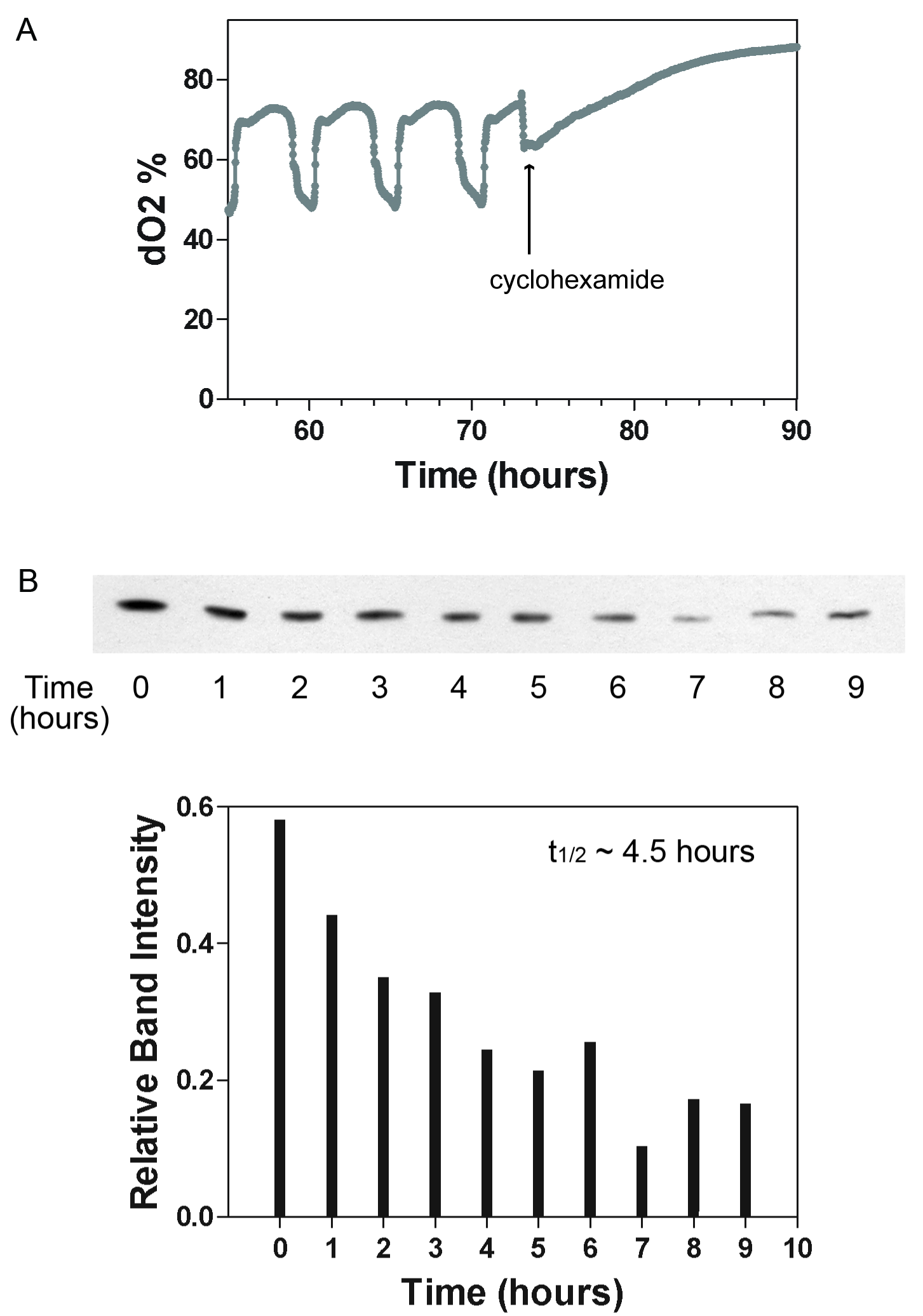

Supplement: Figure S2 — Half-life of GFP in the continuous culture conditions used to observe the YMC. (A) 40 µg/ml cycloheximide was added to metabolically cycling diploid yeast cells expressing pATO3-GFP at the time point indicated. (B) Equal amounts of cells (10 OD600 units) were collected at each time point indicated, lysed in sample buffer (with protease inhibitors) by bead beating, and subsequently resolved and detected as described in the Materials and Methods section. (C) Band intensities corresponding to GFP protein levels were quantified from the blot image using the ImageJ program, and the GFP half-life under these conditions was calculated from this data. The half-life for mCherry (for an mCherry reporter, pDBP2-mCherry) was nearly identical to that of GFP (not shown). The cycloheximide treatment was irreversible and stops metabolic cycles, and the cells in the chemostat did not recover. (0.83 MB TIF) [file pone.0012595.s002.tif]

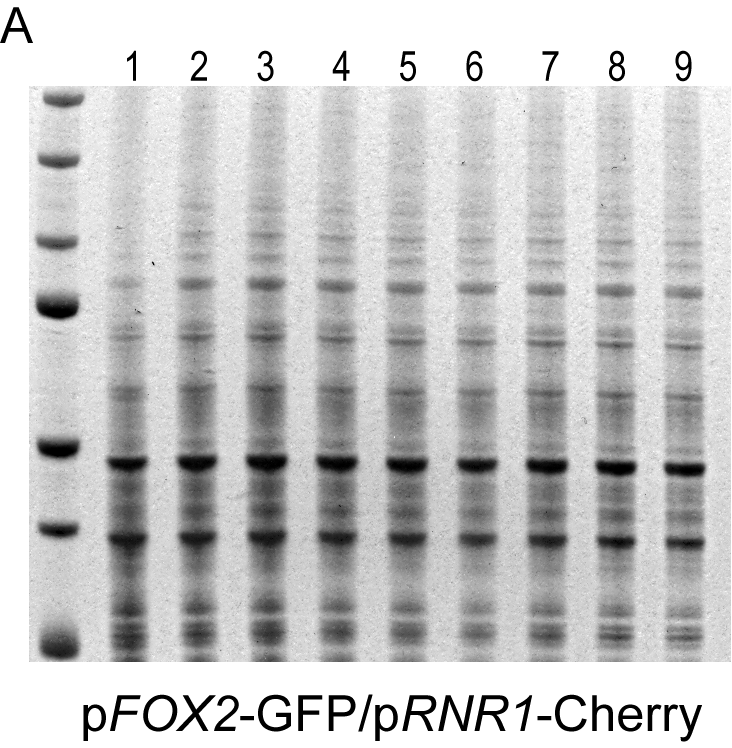

Supplement: Figure S3 — Loading control for Figure 6B. Coomassie-stained gels loaded with equal amounts of the samples shown in the gels/western blots displayed in Figure 6B, obtained from quiescent batch cultures of yeast cells expressing GFP/mCherry reporters. (1.10 MB TIF) [file pone.0012595.s003.tif]
